# The Interactive Management of the SARS-CoV-2 Virus: The Social Cohesion Index, a Methodological-Operational Proposal

**DOI:** 10.3389/fpsyg.2021.559842

**Published:** 2021-08-02

**Authors:** Gian Piero Turchi, Marta Silvia Dalla Riva, Caterina Ciloni, Christian Moro, Luisa Orrù

**Affiliations:** Department of Philosophy, Sociology, Education and Applied Psychology, School of Psychology, University of Padua, Padua, Italy

**Keywords:** interaction, COVID-19, dialogical science, social cohesion, community, emergency, public health

## Abstract

This contribution places itself within the emergency context of the COVID-19 spread. Until medical research identifies a cure acting at an organic level, it is necessary to manage what the emergency generates among the members of the Community in interactive terms in a scientific and methodologically well-founded way. This is in order to promote, among the members of the Community, the pursuit of the common aim of reducing the spread of infection, with a view to community health as a whole. In addition, being at the level of interactions enables us to move towards a change of these interactions in response to the COVID-19 emergency, in order to manage what will happen in the future, in terms of changes in the interactive arrangements after the emergency itself. This becomes possible by shifting away from the use of deterministic-causal references to the use of the uncertainty of interaction as an epistemological foundation principle. Managing the interactive (and non-organic) fallout of the emergency in the Community is made possible by the formalisation of the interactive modalities (the Discursive Repertories) offered by Dialogical Science. To place oneself within this scientific panorama enables interaction measurements: so, the interaction measurement indexes offers a range of generative possibilities of realities built by the speeches of the Community members. Moreover, the Social Cohesion measurement index, in the area of Dialogical Science, makes available to public policies the shared measure of how and by how much the Community is moving towards the common purpose of reducing the contagion spread, rather than moving towards other personal and not shared goals (for instance, having a walk in spite of the lockdown). In this index, the interaction between the Discursive Repertories and the “cohesion weight” associated with them offers a Cohesion output: the data allow to manage operationally what happens in the Community in a shared way and in anticipation, without leaving the interactions between its members to chance. In this way, they can be directed towards the common purpose through appropriate interventions relevant to the interactive set-up described in the data. The Cohesion measure makes it possible to operate effectively and efficiently, thanks to the possibility of monitoring the progress of the interventions implemented and evaluating their effectiveness. In addition, the use of predictive Machine Learning models, applied to interactive cohesion data, allows for immediate and efficient availability of the measure itself, optimising time and resources.

## Introduction

The span between the end of 2019 and the beginning of 2020 saw the global diffusion of the infection caused by the new SARS-CoV-2 virus (Coronaviridae Study Group of the International Committee on Taxonomy of Viruses, 2020). It is characterised by its high diffusion, speed, and easy mutation. This last aspect makes it difficult to know the mutation pattern of the virus' RNA in order to identify an effective therapy system. It is not possible to explain how the virus changes (Giovannetti et al., 2020), what its incubation span is, the duration of the infectious period (Anderson et al., 2020), or the difference of symptoms between infective people (Chen et al., 2020; Lu et al., 2020). While waiting for medical research to identify effective ways to treat and prevent COVID-19 at an organic level, the spread of the virus becomes an opportunity to make a scientific analysis of what is generated interactively in the human community, the *Communitas*, in terms of public health.

*Communitas* is in fact defined as the mass of interactions in continuous change, triggered by members of the human species who inhabit and live in a certain dimension (geographical-territorial and/or virtual), towards the incessant search for a common and shared goal (Turchi and Cigolini, 2017). Currently, a shared aim involving the world population, the *Communitas*, is reducing the contagion spread of COVID-19. Such an aim, then, involves and requires accountability to each species member, enhancing the social cohesion of the whole community.

How can the scientific world contribute to the management of human interactions in an emergency situation, such as the current pandemic, in pursuit of the common purpose of public health, in terms of Social Cohesion?

The necessity, in scientific terms, lies in the need to promote a shift from the use of deterministic-causal criteria to the methodologically valid management of the uncertainty of interaction: this makes it possible to pursue the common goal, which currently concerns the whole human species, in parallel with research and action at the medical and organic level, and also to consider and manage changes in the interactions themselves in the future, when the medical emergency will be over. This contribution therefore proposes a fundamental shift from what science currently makes available to the Community to manage interactions in an epistemologically and methodologically well-founded way. From the common perspective of reducing contagion, interaction is the cognitive process at the basis of the scientific panorama (and not only of the exchange or communication act): *interaction*^1^ is the perpetual process in which two or more elements, in uncertainty, generate more or less stable interactive arrangements, which in turn are subject to changes (Turchi and Gherardini, 2014a).

Such interaction setups, constantly changing, always tend towards, at different degrees, the Social Cohesion of the Community. This, within the theoretical and epistemological framework we are outlining, is defined as the “whole of the modalities, at discourse level, configuring realities that concur to the shared management among the Community members of the key aspects anticipated, thanks to common goals.” (Turchi and Cigolini, 2017). The above definition, in terms of analysis of the Community members' interactions, allows for the availability of interactions measurement tools, including the Social Cohesion Measurement Index. The latter offers scientific data to calibrate the construction of the COVID-19 emergency management modalities on the interactions of the Community itself. The Social Cohesion Index, specifically, permits to obtain a measure of it in terms of the modalities with which the population narrates the current medical emergency situation, allowing researchers to consider the extent to which narratives on Covid-19 are oriented to the protection of citizens' own interests, rather than the promotion of public health. Such an index offers then a scientific datum that can be used to gauge the COVID-19 emergency management modalities construction based on interventions on the Community interactions. Therefore, in the absence of data describing what is happening at an interactive level, the strategies employed to manage the medical emergency risk are random, and to be subject to the personal opinion of the ones fielding them. This bears the risk that the key aspects, found within the interaction configurations, are not managed, and in turn contribute to the fragmentation between the members of the Community, leading to a lesser cohesion for the pursuit of the common goal. Having the discursive data of Social Cohesion available allows, therefore, to enhance the contribution of each citizen, using it for the sake of “Public Health.” Thus, the interactive contribution of the *communitas* citizens allows the promotion of community cohesion and the accountability towards a common goal. To this purpose, there are many scholars who have set out and defined the accountability phenomenon, such as Zuliani (2006), who sets out eight stages people undergo due to a natural disaster. One of these is the so-called “honeymoon stage” and another one is the “hero stage” where citizens help each other in a team and there is a climate of optimism.

In order to deepen the idea of “contagion reduction aim,” it is necessary, first, to distinguish between what concerns the organic medical dimension of the individual and what concerns the interactive dimension of the Community in fundamental terms.

In the course of the historical and philological contingencies, the use value of “Health” has been exhausted within medical care, so that Health has assumed the meaning of “condition of well-being” on an organic level, and medical care has been understood as the integrity of the functional anatomical unit, i.e., the body (Turchi and Vendramini, 2016). In this way, Health is reduced, on a methodological and operational level, to the intervention on the element that determines the pathological condition of the individual, losing sight of the interactive dimension of the community collective aspect of Health. The distinction between Soundness and Health is made in terms of epistemological, and not only semantic, foundation^2^: Sanity refers to the physical and biological integrity of the individual organism, while Health is based on interactions between the members of the Community. Therefore, a criticality is identified, in terms of repercussions on the management of interactions, in the superimposition of foundation and epistemological plans, distinct in the conception of Soundness and Health, which in the current emergency scenario is undermining the modalities of medical emergency management.

If the aim is to contain the spread of the virus and safeguard the capacity of the health care system to respond, as a common good, research on the virus in medical terms is one way of pursuing the purpose. This does not, however, subsume the strategies with which to manage the repercussions already in place on the interactive level (economic, psychological, cultural, social, etc.) that the emergency itself generates, also in terms of changing interactions between members of the Community after the medical emergency is over.

The epistemological distinction of the words thus enables action to be taken in foundational-methodological terms in the management of the impact at Community level towards the common purpose. In the face of praxis that are placed in an exquisitely sanitary dimension of care of the biological organism of the individual, the diffusion of COVID-19 shows how a series of strategies implemented by the population on the interactive level of the whole Community is developing (for example, the neighbour who makes himself available to shop for the condominium's elders). As with the various medical emergencies that have emerged over the course of human history, the virus has forced the human species to modify its interactive modalities, in order to compensate for their decrease against the quarantine and social distancing measures: it is on the level of interaction (therefore not only on that of research at an organic level) that the human species can effectively pursue the common goal of reducing the spread of the virus. It is on this level that the COVID-19 emergency, as far as it can be managed on the organic-sanitary level, will generate changes in the way members of the Community interact: for example, the “fear of contagion” may cause some members to maintain the rule of social distancing even after the emergency has returned. It may therefore change the value attributed to what before the emergency was considered certain and taken for granted: for example, being able to go on a trip, to see a loved one, to have an internet connection at home.

The current impact of the COVID-19 emergency in the community has highlighted how the interaction between the many elements that characterise events in human history reveals the uncertainty of their evolution. The interactive elements of the current world situation can therefore be placed within an uncertain panorama, of which the virus itself is a manifestation: this Principle of Uncertainty (Heisenberg, 1963, 2015) shows how it was not possible to predict the phenomenon of COVID-19 and the extent of its effects on Health, in terms of interactions.

By adopting uncertainty as a Principle, described as the non-possibility to predict future events because the very starting conditions are uncertain, the current situation configures itself as an emergency situation. The emergency in fact is “everything that is produced as a follow up to an event that can to some extent change the arrangement of a community, sticking to a purely descriptive plan of what happens” (Turchi et al., 2015).

Considering this Epistemological Principle as a scientific basis, the below describes how the configuration of the current pandemic presents fundamental and methodological criticalities to be considered and on which to intervene on a management level. This allows one to direct oneself towards the promotion of Community Cohesion on the whole, through referring to interaction measurement data that can support the spread reduction of SARS-CoV-2 virus.

## Promoting Social Cohesion as a Community Need

The scientific panorama wants to trace the knowledge of the virus (Santosh, 2020) through the continuous collection of epidemiological data, in order to identify a constant characteristic of the virus. In monitoring changes in the epidemic, through databases that provide an overview of the epidemic situation (Li et al., 2020), it emerges that the data collected on an epidemiological level are constantly changing, varying according to the study and the elements taken into consideration (Anderson et al., 2020). For example, the WHO considers that the main transmission pathway is through close contact with symptomatic people; however, it does not exclude that it can also happen with asymptomatic people, claiming their rarity on the basis of the frequency of collected data (Istituto Superiore di Sanità, 2020). These aspects make the criticality of fragmentation of the epidemiological data collected traceable, given the diversity and uncertainty of contextual and environmental characteristics that differ from country to country and from individual to individual.

The collected data, although uncertain, are used to make estimates and forecasts of the epidemic trend on which to base political and economic choices for the management of the fallout of the emergency, through the use of mathematical-statistical models taken as reference (Consiglio Nazionale delle Ricerche, 2020). From a clinical psychological point of view, the research panorama highlights a series of possible psychological disorders (depression, anxiety, stress, psychiatric syndromes, etc.; Asmundson and Taylor, 2020b; Brooks et al., 2020; Loveday, 2020), also labelled *ad hoc* (Coronaphobia; Asmundson and Taylor, 2020a), which it considers to be the direct consequence of quarantine and isolation measures. The same happens within social psychology, which offers data on bias, stigma, and racist processes towards the Chinese population and between individuals in the same community (Chung and Li, 2020; Wen et al., 2020). These statements are based on studies carried out through the use of statistical data (Wang et al., 2020) taken as reference in order to explain what the COVID-19 emergency causes among members of the Community. The same data, in this way, is looked at and used through lenses that are always different, so that they will always give different results, even if they are used in order to predict what will happen in the future. Once again, psychological research focuses on human behaviour as an element to be considered in order to determine the spread speed of the virus (British Psychological Society, 2020). Such behaviours, however, remain subject to the uncertainty of interaction, so it is not possible to predict whether the behaviour under study will be implemented, how, and what its impact on the spread of the pandemic will be. Recalling the Principle of uncertainty of interaction, which generates temporarily stable and subject to change settings, the criticality can be traced in the foundations of epidemic management choices based on deterministic predictions, built on continuously changing medical outcomes. In this way, the management modalities do not take into account the uncertainty given by the interaction of the contextual elements of the data base offered, predicting what will happen on an interactive level. Predicting what will happen in managing interactions decreases the social cohesion degree. The prediction alone defines with certainty what will happen after a series of events. Thus, it does not allow individuals to design alternative strategies to handle unexpected realities. Anticipating, instead, a range of possible interactive key aspects that could happen in the community, would allow to manage at the same time, in a cohesive way, the whole community. Social cohesion is the chance that every citizen contributes his/her role in the community, through anticipation (instead of prediction) of the possible key aspects and management strategies, aimed at the pursuit of a common goal. On the contrary, setting out a single future scenario, instead of outlining at different degrees the possible ones, allows to reify what was predicted instead of managing what can happen for a common goal.

For example, when the quarantine isolation of citizens and the closing of commercial and economic activities are decreed by law (WIlder-Smith and Freedman, 2020) it is not possible to predict (and therefore establish future certainty) that a part of the citizenship will lose its job or receive no income from its commercial activity. However, this may still happen. When such management is anticipated and carried out, in the light of each community member taking charge of the common goal pursued, other than the exclusive personal aim, the community cohesion degree rises, and the community will not get fragmented. This, in turn, increases the chances to find solutions and strategies previously not available. These are interactive realities that enable, by the previous example, to find other ways to receive income from one's own business or finding another job; differently, such modalities would not allow safeguarding not related to the citizen. So, where the citizen loses his/her job and does not have any income, in the employment of the sole prediction of what will happen, and thus in the decrease of the interactions, he/she can configure himself/herself as a “desperate person, with no other chances to live with,” contributing to the interactive use of such a label to the fragmentation of the community.

Thus, basing interactive management choices in terms of prediction leads to an array of interactive fallouts that fragment community cohesion, increasing the chance of a rise of conflict creation and more fallouts, of which we offer below some examples. Adherence to the regulations, for managing the spread of the virus (quarantine, isolation, closure of economic, cultural, and educational facilities, etc.; Maxwell et al., 2020), as well as to the directives for the use of protective devices (World Health Organization, 2007, 2020), interactively eludes the regulations requirements: law decrees are becoming every day more coercive or connoted by the “prohibition” mode rather than the descriptive one of possible actions linked to the virus reduction aim. The Community is therefore not made part of the management but is continuously considered to be the mere executor of the restrictions imposed. This contributes to the making of a fragmented community, creating conflicts. Specialised literature unveils that having a third, superordinate aim, in the event of dangers for the whole community (see natural emergencies or catastrophes) where everybody can give his/her contribution and participation, reduces conflict generation (Sherif et al., 1961). Thus, by taking responsibilities for the sake of each member, Community Health becomes the chosen strategy to promote the social cohesion aim (Bifulco and de Leonardis, 2005). The above-mentioned process enables, also in methodological terms, to overcome the aggregation of individual preferences (and thus of the personal aim), towards a more general look, oriented to the Community (Boltanski and Thevenot, 1991).

All this becomes possible if the pandemic “emergency” reality is not considered as an entity, detached from the observer, but as created by the interactions among the speakers that narrate and build the meaning (as for catastrophic reality, see Berger and Luckmann, 1969).

Orienting the Community towards a common purpose then becomes necessary, when the interaction between the members of the species cannot be completely “segregated,” precisely because it is related to a plan of uncertainty and randomness, and not organic determinism. In fact, adherence to the prescriptions is promoted by relying on the moral values of the individual and her or his “motivation” (Anderson et al., 2020) through information campaigns, and not on the basis of a scientific, epistemologically founded method of interaction management. We therefore leave it to the individual to interpret the information he or she receives, thus opening up the possibility that, despite quarantine, the citizen may decide, for example, to have dinner with friends, without him or her contemplating the possible repercussions that this action will have on the national health-care system, and therefore on the Community as a whole. Since the citizen is not adequately trained in the management of virus transmission, he or she relies on his or her own beliefs and opinions on the extent of the emergency, which are different from those of other members of the Community (see in this direction the propagation of fake news, the increase of controversy and contradictory interpretations of regulatory bans, comments offered to the social pages of institutional representatives, etc.). In this way, the fragmentation of the Community's interactive management modalities jeopardises the conservation of the species. Fragmentation focuses mainly on meeting the needs of the individual (“I need to get out and walk, I'm going for a walk”) and not on the common need of its members (“I could create a voluntary service that would allow the families of COVID-19 infected people to stay in contact with them, however isolated they may be in a hospital ward”). In the face of these examples, the fragmentation of emergency management strategies, built hic et nunc, without scientifically basing the operational mode of scenarios that could be effective for the management of interactions, is critical. This may affect the possibility of the population moving in a cohesive manner, towards the pursuit of the objective of virus reduction, thus the very effectiveness of the interactive repercussions of the medical emergency management process. Consider, for example, the increase in infections following the issuing of the Italian Prime Minister's Decree amending even slightly the previous provisions: “use of suitable devices” and “it is possible to play sports near your home” is interpreted as “then I can go jogging as long as I wear a mask and cover my mouth and nose when I meet someone.” Given the above definition of *Communitas*, the pursuit of this common objective will in fact be all the more effective the more interactions between the members of the Community will increase, in the construction of emergency management modalities, towards *Social Cohesion*. Doing so makes it possible to promote, among the members of the Community, competencies enabling them to take on the role of citizens capable of contributing to the pursuit of the common objective. Managing in a shared way, therefore, what happens in the face of the medical emergency, brings with it the need to identify the critical interactive aspects of the situation in which you find yourself. Citizens can thus be put in a position to act on the problems anticipated, in synergy with political and administrative institutions. In this sense, each one is a node within a network of interactions, therefore able to manage what happens, sharing the aim of reducing the spread of the virus. This can be observed, for example, in the part of the population that has mobilised to build alternative oxygen masks to those used until now in the medical sector, starting from the criticality found in the availability of oxygen masks in hospitals.

## The Dialogical Science

The Principle of Uncertainty of Interaction, and the construct of Social Cohesion, therefore become useful references for structuring and implementing ways of managing what happens interactively, in the period of medical emergency. It becomes necessary, at this point, to place oneself within a scientific panorama capable of building knowledge from interactions.

Just as it is not possible to perform a surgical operation without any anatomical knowledge, in the same way it is not possible to manage what a medical emergency generates at an interactive level, between the members of the Community, without any knowledge of the uncertainty of the interactions that configure the emergency reality, with repercussions in terms of its Health.

It has become necessary to move towards the explicit dimension of scientific sense (Wittgenstein, 1999; Turchi and Celleghin, 2010; Turchi, 2002) which offers a common and well-founded basis, from which to build effective ways of emergency management, in interactive terms.

The proposal for interactive repercussions management described below is placed within a *conceptual* realism^3^, in which the interactive reality of *Communitas* does not exist in itself but is built in the act of knowing it. Referring to the uncertainty principle of interaction, it is not possible to know the state of reality, but rather the process of its generation (Turchi and Vendramini, 2016), which is thus linked to “how” it is configured, and not to “why” the cause for which it exists in itself.

Therefore, starting from the shift from mechanistic paradigms to interactionist paradigms (Khun, 1962; Salvini, 1998; Marhaba, 2002; Turchi et al., 2007; Salvini and Salvetti, 2011), in order to be able to base the choices of world emergency management on scientifically based data, the proposal of this paper takes shape in Dialogical Science® (Turchi, 2009; Turchi et al., 2012b; Turchi and Gherardini, 2014b; Turchi and Orrù, 2014; Turchi and Vendramini, 2016; Turchi and Cigolini, 2017; Turchi and Della Torre, 2017)^4^. This takes charge of the discursive or dialogical process of knowledge (Turchi, 2013): it is therefore placed within the incessant flow of a process, which every time it is shown takes shape through contents. Content is configured as, precisely, “what it contains,” the product, which is or is not there, of a certain procedural dimension, always present (Turchi and Celleghin, 2010). For example, government action to make masks available, free of charge, to residents of an Italian region, remains a content, a product, which is linked to a process of managing the emergency fallout in the Community. The process, in this case the management, does not stop, and takes shape in the specific contents, the actions that are implemented: in order to manage what happens in the Community at an interactive level, it is then also possible to make available a bonus for babysitter salaries, hospital construction, and fundraising for the healthcare system. This is due to the uncertainty of the product or content, so that the form the process may take varies continuously based on the interactions within the Community. The interactive-dialogical process therefore generates configurations of realities (Turchi, 2013) with variable stability through the interaction between rules^5^ of use of ordinary language.

Ordinary language^6^ (Wittgenstein, 1964, 1976, 2009; Foucault, 1967; Gadamer, 2000) is the object of investigation of Dialogical Science, as a generator of reality configurations of all those who belong to *Communitas*. The discursive reality thus can be described in terms of process, starting from the rhetorical-argumentative links that describe it as such (Bruner, 1991). The conceptual shift occurs, therefore, in considering *Communitas* not as a set of individuals but as a set of discursive productions that interact to generate discursive reality arrangements for the management of the medical emergency. How can management choices, based on medical data, take charge, scientifically, of discursive reality configurations?

**Figure 1:**
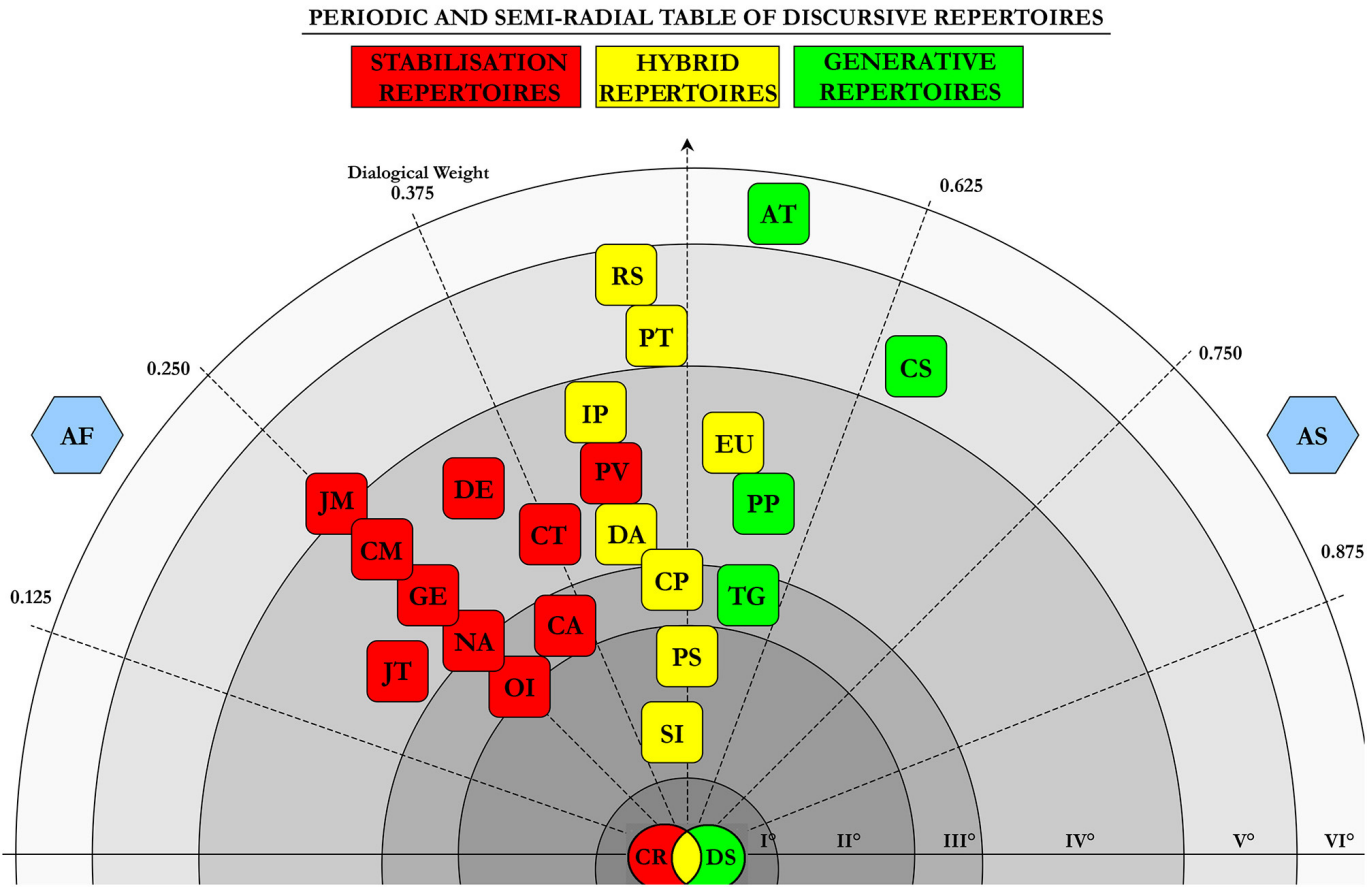
Periodic and Semi-radial table of discursive repertories.

The Dialogical Science research program allows to generate knowledge about interactions thanks to the formalisation of the rules of use of ordinary language. A formal language is built (Turchi and Orrù, 2014) out of the ways in which ordinary language is used in interactions, thus describing the ostensible property^7^ of language. The formalisation of the interactive-discursive rules allows to obtain scientific data of a discursive type: this is possible by establishing, a priori, the rule of use of ordinary language. The configuration of sense of reality that is generated, in the use of these rules, is already defined a priori, regardless of the use of the language itself. Changing the rule, therefore, changes the sense of reality that is generated, and therefore the data that is offered (Turchi and Celleghin, 2010). This is also what happens within mathematical algorithms: by changing the value of the symbolic units used, the product of the algorithm will be different from the previous one. In the formalisation of the ordinary language, the same change occurs in the sense of the configuration of reality, generated by the data offered by its measurement. These data are in fact organised in the 24 Discursive Repertories (R.D.)^8^, available in ordinary language to configure meaning reality. According to the studies conducted in this field (Figure 1 and Annex 1—Periodic and Semi-radial Table of Discursive Repertories—Glossary), there are 24 possible and available RD for the human species. From research conducted in literature it emerged, in fact, that against a discursive space available to interactive individuals^9^ (that is each of the 24 RD characterised by specific process properties peculiar and distinctive), some RD aggregations of those (Mariotti Culla and Turchi, 2007) outline profiles and degrees of social cohesion. Therefore, against the 24 possible discursive modalities, a cohesion interactive setup is created, whenever the observation of some of those is privileged against other ones (such as the Targeting, Description, Consideration, and Proposal). Vice versa, as for conflict setups^10^, experimental texts have evidenced some modalities among the 24 available, that emerge with less frequency compared to other ones.

The interactions among the R.D. generating discursive configurations are organised within the Periodic and Semi-radial Table of Discursive Repertories (Figure 1), according to their generative properties of sense reality. The Table represents the main guiding tool in the analysis of the properties with which the ordinary language shows itself, and the extent to which the Community is inclined to change, in the management of what the medical emergency generates, at an interactive level. How much, therefore, moves in terms of Social Cohesion, to manage in a shared way and in anticipation of what happens, in pursuit of the aim of reducing the spread of contagion. In particular, as evidenced by the graphical representation of the Semi-radial Table, some modalities defined as maintenance ones (red colour) have been isolated, allowing the creation and maintenance of conflict profiles. These include Certify reality, the Cause of action between the discursive items, Judgement, and Comment. Other modalities, defined as generative ones (in green) represent social cohesion configurations among the interactive individuals. Last, but not least, hybrid modalities have been described (in yellow), that by themselves do not create conflict configurations but when associated with the maintenance ones they speed them up, increasing the degree of conflict exercised by the interactive individuals.

## The Measure: the Generativity and Dialogicity Indexes

Starting from the rules of the formal language it uses, Dialogical Science offers the theoretical-methodological elements able to measure the discursive configuration in a precise kairos (instant, moment). This makes it possible to manage the uncertainty of the interactions that are generated in the Community, in the face of the medical emergency: in view of the measure, it becomes possible to intervene by orienting the interactions themselves towards Social Cohesion. The measurement is offered by assigning to each R.D. two units of measurement: the Dialogical Weight (PD) and the Dialogical Moment (MD). The first offers a measure of the Generativity Index^11^ (Turchi and Orrù, 2014, p. 2), that is “the ability of the Repertory to generate different and multiple discursive configurations” (Turchi and Cigolini, 2017), with a value between 0 and 1; the second offers a measure of the Dialogicality Index, that is the type of link that is created between the R.D., and the “strength” of this link in terms of narrative coherence^12^ (Turchi and Cigolini, 2017). Both measurement indexes describe which discursive modalities interact, and in what way, in the generation of a certain reality configuration. The Generativity and Dialogicity measurement indexes can indeed be measured in anticipation (before the implementation of an intervention), in itinere (during the development of the intervention), and *post hoc* (after the intervention has been completed), presenting scientific data that can be used to pursue the intervention objective that is set in the most effective way.

Thanks to the availability of the measure of the interactions, generated by the *Communitas* members, it is therefore possible to intervene on them. Starting from the measurement indexes presented, it has been possible to build methodological praxis of the dialogical operational model, able to intervene on the interactive events of the Community (Turchi and Della Torre, 2017). Among them the anticipation (and not the certain prediction of a single possibility, as happens on the medical level) of a range of interactive scenarios is scientifically based (Turchi et al., 2013b). These could be described as critical in pursuing the shared aim of reducing the spread of the virus: for example, the possibility that elderly and lonely people may find it difficult to have access to what they need (shopping, medications, etc.), or that some members of the Community may not conform to the legislative requirements. *Anticipation*, a necessary step for the management of critical aspects from the perspective of Social Cohesion, is therefore a methodological practice on which to build emergency management methods, starting from the measurement of interactive data, and no longer only medical data, and the trajectory of narrative coherence traced by the speeches themselves. For example, it is possible to measure the citizen's contribution to the Community when he says that he “feels extremely lonely because he is in need of hugging someone,” so he decides to go out despite the quarantine measure, or else, when he says that “although this situation makes me feel lonely and I am not used to it, it can be an opportunity to think about how to make people in quarantine feel less lonely.” Therefore, in the view of what has been measured, it is possible to anticipate the trajectories of interactive development of both statements, and to calibrate the management strategies of the critical scenarios anticipated, before they actually occur, that can increase the degree of contribution to Public Health and therefore to *Communitas* Social Cohesion. Through the use of anticipation of a range of future possibilities, the members of the Community are in a position to consider more possibilities for the pursuit of the shared purpose. The measure therefore enables these possibilities to be considered in terms of scientific data on which to base management choices of possibilities themselves.

Methodologically, therefore, it becomes necessary to collect, observe, and analyse the text produced by the members of the Community: this makes it possible to measure the configuration of the reality under investigation, through the use of R.D. and measurement indexes. As already presented, on a theoretical point of view, in the previous section, experiments conducted with a control group who were asked to generate conflict setups against a verified actor, it emerged that when a party exercises the cited maintenance modalities, the other party uses in turn the same discursive maintenance modalities, but among all those privileged we find Justification and Certify reality. Also, when conflict setups are described, as those cited in the discursive productions, Evaluation, Description, Consideration, Opinion, and Proposal modalities decrease. That is, the conflict discursive configuration (maintenance, red) erodes the available social cohesion part (generative, green). So, the range of possible interaction setups spans between two polarities, conflict and social cohesion: the more modalities creating conflict are isolated, the fewer social cohesion discursive configurations can be created.

Therefore, each text, produced as regards the study object, and therefore, in this paper, concerning the medical emergency linked to the Covid-19 spread, contributes to the Community social cohesion level. The assumptions regarding how the virus spreads, the stances towards the legislative decrees issued, the moods linked, for instance, to worrying about oneself and the loved ones, and the behaviours and the precautions adopted to diminish the contagion risk, are all considered in the analysis. These all become specific items, with particular content, within the interactive-dialogic process of building reality configurations. All these, to a different extent and with varying force, concur to the generation of a particular community setup creation—more or less cohesively—for the pursue of the virus spread reduction aim. What has been produced by Dialogical Science in terms of measuring reality configurations has been implemented in several research and intervention projects, including a study completed in 2011 in the Italian territory of L'Aquila following the emergency generated by the natural catastrophe of the earthquake of April 6, 2009. The collection and analysis of the text, through study protocols with open questions to 2000 inhabitants of the territory affected by the earthquake, measured Health indicators of discursive interactions, and therefore of social cohesion, in terms of citizens' competence in the management of the emergency and participation in community life: they draw a description of how the participants configure their social and interactive reality in terms of Community Health in facing the “catastrophic event” (see Turchi et al., 2015). The collected data showed a configuration oriented to the maintenance of the “catastrophe” reality, as it is, mainly linked more with the use of Repertories such as Certify reality, Opinion, and Judgement, and less to Repertories such as Description or Targeting.

Data allow then the implementation of public policies that place citizens in the perspective of shared management of the critical aspects generated in response to the catastrophe, thus directing them to the perspective of Social Cohesion. Another application took shape within a 2011 study (Università degli Studi di Padova, 2011) describing the process of discrimination (“stigma”) against people affected by HIV within the Italian territory, conducted on a survey group of 1,267 respondents (among people affected by the virus, family members, health and social workers, and citizens not affected by the virus), who were administered an open questions set protocol. The researchers, through a series of evaluation indicators and the availability of indicators to measure the degree of stigma against people with HIV, have obtained data on how the members of the Community, in their role, narrate the interactions related to the object of the survey. The subjects involved in the study employ maintenance discursive modalities, through narrations like “Every day is a struggle to survive” (Certify reality R), or “It will be hard to achieve a normal family condition” (Prediction R). In particular, the cognitive plan in which we have set ourselves has made it possible to measure the Generativity of the stigma towards people affected by HIV at a national level and the Dialogicity of the narrative coherence of the participants in relation to the construct of the survey. The fluctuation in the value of the process of discrimination therefore provides the starting point for managing stigma within the Community, intervening on the interactions and roles involved more or less directly with respect to the issue towards a shared management of this critical aspect, therefore in the perspective of Social Cohesion.

A final example of what has just been described is the InOltre Service, an essential level of assistance within the Veneto Region (Italy) also based on the assumptions of Dialogical Science. The service was born in 2012 with the aim of “promoting the Health of the territory through the management of the implications of the socio-economic asset” (Turchi and Cigolini, 2017; Turchi et al., 2019). The availability of a measure of the interactions and the reach of the objective described above make it possible to take charge of the repercussions of emergencies at an interactive level in anticipation: this is done in terms in which it allows flexible intervention, depending on the changes that are generated in the Community, directing users towards the pursuit of the common purpose in the management of critical aspects in a shared way. Thanks to the use of the anticipation practice, it has in fact been possible to extend the service, over time, from entrepreneurs in crisis or bankruptcy, to savers victims of the banking crisis, to citizens in critical biographical moments, to Italian citizens during the emergency COVID-19. Taking charge of the user base is made possible and effective by the availability of text measurement indexes on which to base anticipations of the users' narrations, regardless of the content they offer. The text^13^ productions of the service users, that approach the service due to reasons linked to the pandemic, during the phone calls that the service receives and records, could then employ discursive modalities mainly of the maintenance type as Prediction, Judgement, Cause of action, and Comment, offering narrations such as “I've tried everything, I try to fill my days with things to do to keep well during quarantine, but in any case I feel bored” (Certify reality R. + Contraposition R.). The use of Hybrid Repertories, such as Possibility (“I could take advantage of the quarantine to get a new hobby”) could also be collected.

The measurement indexes obtained in the applications described above therefore make it possible to have available a datum related to whether or not the Community's narratives are open to different discourses on the configuration of the reality under investigation (whether it is an “earthquake,” “banking crisis,” “HIV,” or “COVID-19” emergency). The datum makes it possible to describe the Community members' discursive modalities and to evaluate the direction in which they are oriented. The more interactions increase and are directed towards the construction of alternative sense realities to those available, the greater is the possibility that the members of the Community manage critical aspects in a shared way towards the pursuit of the common goal. At public policies level, having available measurement indexes becomes useful to build medical emergency management interventions, specifically gauged against the discursive modalities fielded within the Community and the anticipation of the future community setups. Moreover, it serves to assess their effectiveness and efficiency, both in the short and long term, to increase social cohesion. That is, interventions that take into account, at each stage, the stance and the opinions of the Community members about the medical emergency, the behaviour, and the actions taken for its management (how such discursive modalities are oriented for the pursue of the virus spread reduction common goal in the most cohesive way).

In 35 years of activity, the Dialogical Science research program has conducted research and interventions in different fields, such as psychiatric (Turchi et al., 2016), sports (Turchi et al., 2008, 2010a), social (Turchi et al., 2012a; Iudici et al., 2018b), economic (Turchi et al., 2008), penitentiary (Turchi et al., 2013a), emergency (Turchi et al., 2015), health (Iudici et al., 2018a), community (Turchi and Romanelli, 2019), mediation (Turchi et al., 2010b), geriatric, and engineering, collecting, about 200,000 fragments of text. Now, thanks to its theoretical-methodological structure, the research program has also been able to be inserted within the medical emergency of COVID-19. Over the course of time, the research program has taken the opportunity to have the interactive measure of the reality narrated by the members of the Community available in an almost immediate way, thanks to Machine Learning technology (Turchi et al., 2020). By using Machine Learning technology, it is possible to increase the capacity of text analysis through the methodology used in Dialogical Science, thus being able to deal with Big Data on specific geographical or thematic areas/contexts/areas. The output data of the analysis carried out through Machine Learning allow to obtain and manage different and uncertain discursive configurations in their manifestation, through a predictive model that identifies the Discursive Repertories used in the text, so as to be able to measure the indexes and subject of the search, contemplating and calculating the margin of error of the measurement itself. This is made possible thanks to the use of Artificial Neural Networks (NNs), enhanced by the use of particular NNs, i.e., Recurrent Neural Networks, designed to accept vector sequences as input and produce, as output, and as integrations of the entire sequence submitted to them. This makes it possible to name the Discussion Repertories in a digital way.

What has been described allows researchers to intervene on the emergency reality available from computerised data that do not stop at the logical-grammatical analysis of the text but offer an overview of how the language configures reality, optimising time and resources. In the critical interactive emergency situation of COVID-19, this enables the quick and efficient availability of the interactive scientific data needed to calculate the Social Cohesion Index. The above enables the political-management roles to set up and structure management modalities precisely gauged to the interactive “status” of the Communitas members, as for Public Health in the current medical emergency period (Heymann and Shindo, 2020). For instance, it is considered how the extended use of protective masks during the social distancing period has been perceived, against its effectiveness and the effects it has on people, or else, the various opinions, either expert-like or not, related to the present and future outcome of the pandemic on physical and mental health and on interpersonal relations. In this sense, to have this kind of data available, precisely gauged and quickly analysed, and therefore always up-to-date and pertinent, enables the management roles previously mentioned to monitor, assess the activities, and choose the initiatives and policies implemented in the territory tailored for the citizens, both at the time of measurement and with a future perspective, thus promoting social cohesion of the Community and reaching the highest effectiveness and efficiency levels.

## The Measure: The Cohesion Index

Starting from the theoretical foundations and the formal language of interaction, Dialogical Science makes available the immediate processing of interactive data, on which to base management choices at the level of public policies: there is a measure of how *Communitas* citizens are using the interactive modalities available to them in relation to emergency management and how they are moving in pursuit of the aim of reducing the spread of the virus, whether or not this is in a cohesive manner. In this direction, the progress of the Dialogical Science research program has made the index measuring Social Cohesion available.

As we previously saw, the Community aim during the Covid-19 pandemic is the contagion spread reduction. Considering this context item, i.e., a text item, concurring to (is part of) the studied discursive configuration, we asked ourselves what is the use (the value) of what the Community offers (in terms of discursive productions) for the defined object^14^. The study question for the measurement of the social cohesion level is then: what is the use value of the discursive configuration observed, against the common goal of the reduction of the contagion spread? The index shows how much the discursive configuration of the Community member is oriented towards the management of critical issues in the pursuit of the shared aim or, vice versa, how much it is moving away from it, thus moving towards the fragmentation of interactions, which can affect the pursuit of the common objective. In order to make the Cohesion construct measurable, the two dimensions in which it has been declined are the anticipation of future scenarios^15^ (variable x) and shared management (variable y), the two variables that are constantly changing according to the objective to be pursued. The common goal is always present and may vary depending on the context.

Once again, the Discursive Repertories are configured as the elements able to offer a measure of Cohesion, in relation to the role they play towards the two variables defined above. It has therefore been defined which Repertories, and to what extent, are included within the definition of Social Cohesion, in its dimensions of shared objective, anticipation of critical aspects, and shared management of the same (see definition on page 1).

It is assumed that the use of some Repertories contributes to the pursuit of the common goal, the use of others less so. The Repertories, by interacting with each other, make a degree of cohesive contribution that varies according to their use in pursuit of the common goal of reducing the spread of the virus. If the discursive elements are aimed at pursuing different objectives than the one set, then the degree of cohesion is assumed to decrease. Conversely, these will increase it.

In order to offer a measure of how it contributes to Social Cohesion, each Repertory of the Table is associated with a value, the “Cohesion weight,” expressed in positive or negative terms. If expressed in negative terms (for example, the Repertory of Judgement, associated with the value −2), it always contributes to a decrease in the degree of Cohesion (as in the following text: “Council members are by no means available and Police forces are sometimes not collaborative enough with citizens”). If expressed in positive terms (for example, the Repertory of Description, associated with the value +9), it always contributes to an increase in the degree of Cohesion (as in the sample text “The neighbourhood population consists mainly of elderly people and students”). If the symbol associated with the value is ±, the Repertory may or may not contribute to the degree of Cohesion depending on the orientation to the objective set (as in the text: “All this chaos in the city must be cleared”—Certify reality's Repertory, configuring a stagnant and unchangeable reality).

**Figure 2:**
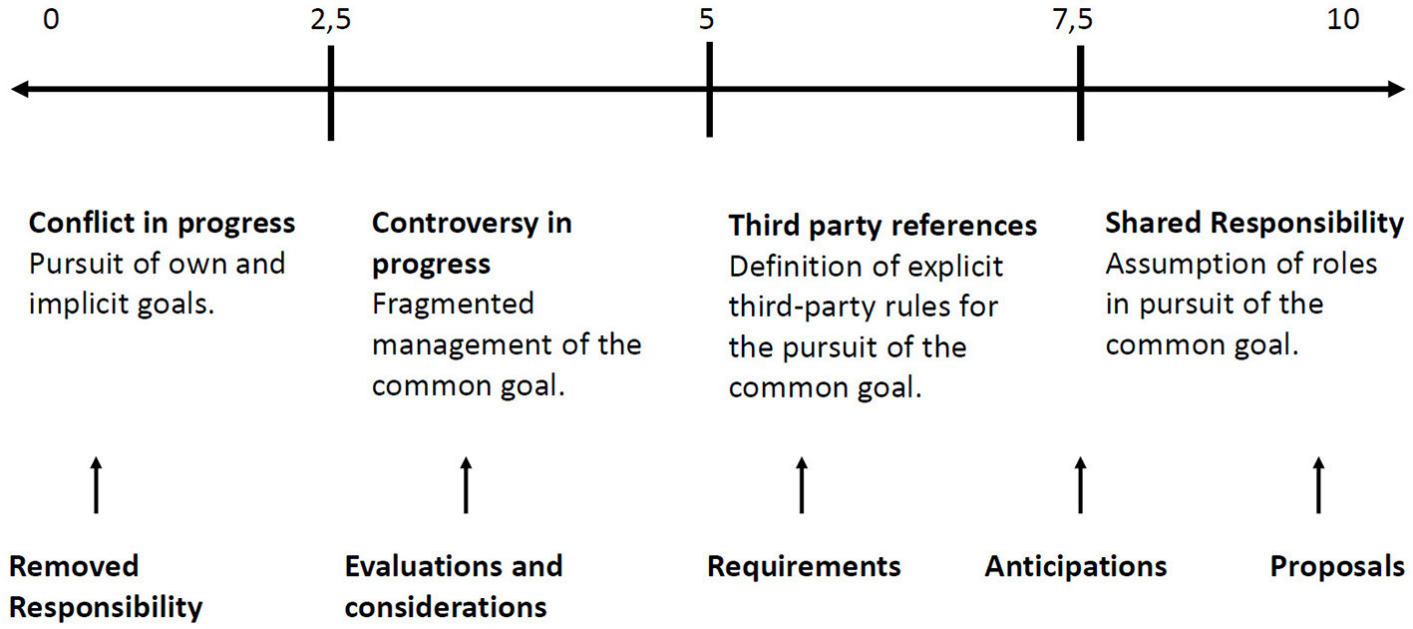
Cohesion continuum.

By associating the Discursive Repertories with the variables in which Social Cohesion is declined, they are considered in increasing order, according to the degree to which the properties of the R.D. themselves contribute or not to the variables, and therefore in a wider sense, to Cohesion. The Anticipation Repertory is one that increases Cohesion to the highest degree (“Considering how the number of infections is decreasing, the government may decide to relax the containment measures by making specific provisions with which each business can re-open”). The Proposal Repertory is one of those increasing the variable of shared management to the maximum degree (“Since we cannot spend Easter with our loved ones, we can have a party among us tenants and involve family members online”). In this way, due to the R.D. used in the analysed text and their Cohesion weight, the variables oscillate within a continuum from 0 to 10 (Figure 2) which accounts for how the Community is moving to reduce the spread of the virus (i.e., the degree of Social Cohesion), through the output data resulting from the application of the index itself. It is thus possible to describe whether the Community narratives are pursuing the objective of Cohesion in terms of shared Responsibility^16^ or whether they are generating fragmentation, pursuing personal and implicit goals.

Getting to the core of this last-mentioned aspect: since Discursive Repertories' Dialogic Weights give evidence of the capacity to create “other” discursive configurations, different from the one already available in the dialogic process, a discursive configuration with high Dialogic Weight (Turchi and Orrù, 2014) makes possible in the language use an interactive movement aiming at the pursue of the set aim. At the same time, considering also that each Discursive Repertory is linked to the other ones according to the Dialogicity Index, the Dialogic Moment, a low degree of discursive configuration Dialogic makes a low capacity of the Repertories to create relations, indicating that the dialogic process can only minimally change (see the Periodic Semi-radial Table); a high degree of Dialogicity, instead, evidences a discursive configuration where the Repertories come more into relation with each other, therefore they get together making more convertible (high ostensive value) the attribution of the use value of a reality status (including the one set as the defined aim).

Hence, a high Weight and high Dialogic Moment is an indicator (in the uncertainty assumed as a Principle) of a high range of possibilities to generate reality sense with a high ostensive value; this brings a high uncertainty degree of the dialogic process trend, even against the adherence chances to the Targeting. A low Dialogic Weight and low Dialogic Moment configuration indicates, instead, that there are not many chances to generate reality sense (and therefore of adherence to the Targeting); such chances produce configurations that depict a reality sense, at high maintenance and exclusive level. We can state, then, that the highest degree of adherence of a discursive configuration to the Targeting Repertory^17^ is obtained from the wider (configuration possibilities) range that can be produced by employing ordinary language.

Operationally, we will name the Discursive Repertories of the Table, considering the adequacy of the reported text with respect to the pursuit of the common objective of reducing the spread of the virus. This adequacy is observed in relation to the shared management, i.e., in the definition and adherence to third-party and explicit management rules.

The cohesion output measured by the index is calculated using the following formula:

$$\begin{array}{l} Cohesion\;Index=C=\left(\frac{\Sigma \;dw}{\Sigma \;dm}\right) \end{array}$$

From the formula, the highest adherence contribution to the Targeting Repertory is created from the use of the Description Repertory, while the minimal contribution is produced from the Justification Repertory use. It is by virtue of the formula result that the social cohesion construct continuum is defined.

The results of the analysis of the texts produced so far by this tool offer, for example, the starting point from which to develop intervention projects on interactions between Community citizens, but also the monitoring of the intervention itself, as well as the evaluation of its effectiveness (in the gap between the result before and after the intervention). The data analysis thus becomes employable in parallel with regulatory-institutional management, in order to increase its impact of effectiveness in interactive terms. The Cohesion Index is used within the Social Cohesion Observatory, a University project aimed at sharing with the Community data concerning the measure of social Cohesion of the Community in an emergency situation, while supporting the Veneto Region in its choices related to the pandemic fallout management.

The Cohesion Index, together with the measurement indexes of discursive interactions regarding a precise configuration of reality (see examples above), offers a common lens of observation of what happens interactively as a result linked to the COVID-19 spread medical emergency—for instance, the discursive productions about this world crisis, the actions taken, the adopted behaviours, the found criticalities—the indexes make it possible to have available data that can be shared, on the basis of which the effects of the medical emergency can be taken into account interactively in the Community and their management can be scientifically based. The data offered by the Cohesion Index allows the population, starting from the institutional and political roles and task forces built specifically for the management of the emergency, to have data about how the Community is narrating what is happening: for instance, describing and offering shareable considerations, generalising issues, judging what is implemented in the territory, expressing opinions and points of view as regards the perceived concern, etc. Likewise, it allows to consider what possible developments could meet the configuration of the emergency reality in terms of change and its management, against such employed interactive modalities.

**Figure 3:**
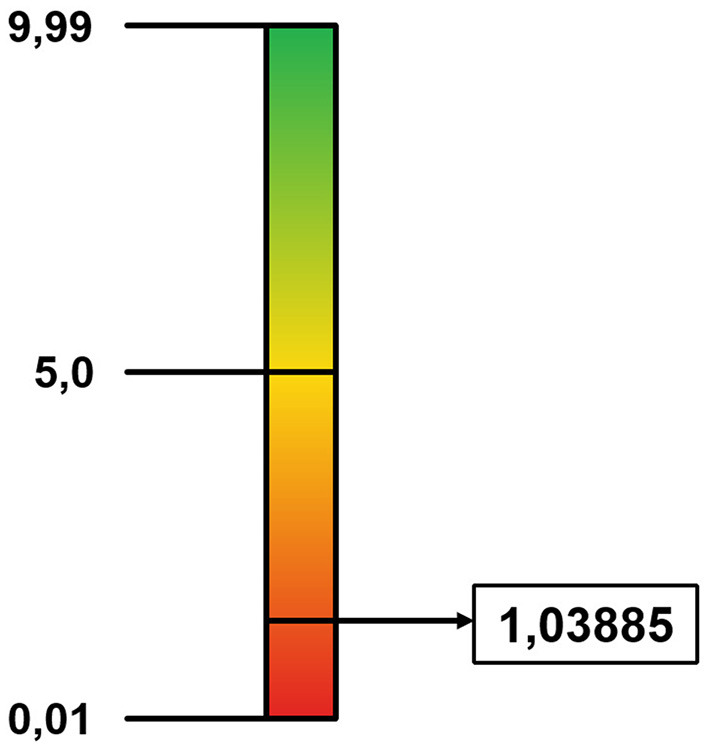
Cohesion value—Santo-Portello District.

**Figure 4:**
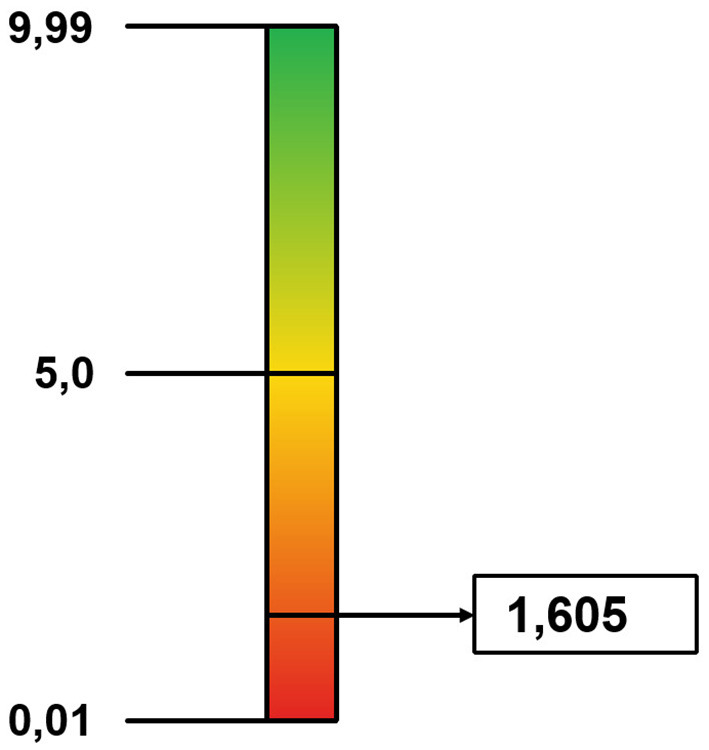
Cohesion value—Arcella District.

An application of the Social Cohesion Index took place in 2019 within the Municipality of Padua (Veneto): within the study conducted, several territorial hubs (citizens, services, businesses, and municipal administration) were involved, including the University. The survey protocols analysed produced data on how respondents narrate their role within the network of interactions in which they are embedded. In both districts considered in the study (Santo-Portello and Arcella), the calculated Generativity gave the output related to the degree of Cohesion: the respondents configure their role according to modalities that keep reality equal to itself, closed to the generation of alternatives (PD equal to 1.03885—Figure 3—and 1.605—Figure 4). This data constitutes what the public administrative apparatus can refer to in the construction of political choices for the management of the Community, to direct the narrations of the population towards the pursuit of a common objective, in view of Social Cohesion.

By decreasing what the Cohesion Index can offer in terms of measurement, it is possible to assert how, in the uncertainty of the interaction, every configuration of discursive reality that takes place in the infinite interactions contributes to generate narratives such as “I am not even sick, I would like to go out to dinner but you can't, it's like being locked in a cage,” but also “considering that from home I can't move for legislative provisions, I could take advantage of it to propose a service for the less well-off in medical emergency situations.” Concerning the Social Cohesion construct and its measurement index, the first text exemplified above offers a personal position of the speaker, oriented to the satisfaction of the personal interests of the individual; the second textual example offers a proposal of a citizen oriented to the management, in terms of Shared Responsibility, of the critical aspects emerged interactively, towards the pursuit of the common goal of reducing the spread of the virus. The measure of Social Cohesion is therefore based on what is made available in the discursive modalities of language.

## Conclusions

The theoretical-methodological proposal described so far, focusing on the Community's Cohesion and Health dimensions, brings added value to the management of the medical emergency repercussions. The value comes real by the measurement, through numerical data, of the interactive setup characterising the Community in this emergency period pursuing the common goal of reducing the SARS-Cov-2 contagion spread. The focus is placed on the dialogic interactive process leading and concurring to the creation of discursive productions, stances, behaviours, and actions related to the current situation where the human species lives. Added value that, again, is found in the chance, thanks to such measurements, to anticipate even the possible scenarios that could happen against the discursive modalities currently employed by the Community members, allowing for more effective and efficient management. This is made possible starting from the epistemological distinction between Soundness and Health, which makes it possible to consider the former within a purely organic panorama of individual body care, and the latter as part of an interactive plan involving all members of the Community: considering this scientific-fundamental distinction and the formalisation of the interactive-discursive modalities made available by Dialogical Science, the proposal indeed offers indications and rigorous tools for measuring and analysing the emergency context, as well as the possibility of monitoring effectiveness evaluations of the interventions implemented within the network of interactions between the members of the Community (Turchi and Gherardini, 2014b). The Cohesion Index also makes it possible to have the measurement of interactions available, offering shared and common data on which to base the construction of what allows management of the critical issues generated by the medical emergency at an interactive level, brought both by citizens and delegates of the various stakeholders. Such an index, in fact, enables the policy makers and the various management roles to effectively move in favour of the Community living in the territory, i.e., promoting the contribution towards a shared and cohesive management of the current emergency. To say it in simple words, having a datum describing and measuring how and by how much the community interacts, cohesively, adhering to prescriptions related to mask use in social distancing situations, enables the above-mentioned roles to adjust and precisely gauge the legislative prescriptions and their communication in order to maximise their efficacy. Moreover, these data enable the political-administrative roles to find management modalities that can be shared with the delegates of the various labour categories, opening up to scenarios that allow keeping the territory and the Community inhabiting it cohesive and Healthy.

These processes are enhanced in terms of efficiency and cost-effectiveness through the use of Machine Learning, which allows you to have the interactive scientific data you need to operate immediately available, including the margin of error of measurement, on a par with mathematical models. The availability of a scientifically founded measure makes it possible to manage the fragmentation of the way in which medical emergencies are managed, shifting them to the dimensions of anticipation (and no longer prediction) and shared management with a view to Community Health and Cohesion. In taking charge of interactions between the members of the Community in times of emergency, it becomes possible to effectively support organic research in the medical field, offering the possibility to operate in the management of what happens interactively in the Community, i.e., what is not on a purely organic level.

In perspective, therefore, public policies are in a position to invest in the effectiveness of scientifically based interventions that support the political-administrative roles in the involvement of the Community to manage what happens within it, enhancing the effectiveness of normative prescriptions. At the same time, an efficiency criterion is met when public policies are in a position to manage, by optimising costs, the resources available in an appropriate and coherent way with the Community's own interactive arrangements. These changes can be found in various contexts, such as education and training (distance learning, through internal platforms), work (smart-working), soundness (construction of new intensive care beds or extended working hours for health workers), transport, economy, and so on. At an interactive level, what is generated in the face of the COVID-19 emergency also concerns the interactions between the members themselves, decreased and modified by the measures of isolation, quarantine, and social distance, and compensated by remote communications (video calls, teleconferences, instant messaging, etc.).

A limit to be considered and to be managed during the application of the index and of the naming method of the discursive Repertories is the human expertise applied to the texts reading and naming process. The naming expertise is taught through *ad-hoc* courses for the application of the naming algorithm, available in literature since 2009 (Turchi, 2009).

Precisely to try to manage this limit, over the last 2 years a research project has been started to measure the human naming mistake, through a collaboration between the FISPPA Department and Padua's Mathematics Department. Such key data for research enables making the current work (and the research) more accurate.

Finally, we anticipate how, once the medical emergency is over, it will be possible to trace the changes that it has generated in interactive terms in the *Communitas*, which can already be described from the narratives of its members. Through instruments for measuring and evaluating the effectiveness and efficiency of interventions, the proposal of Dialogical Science allows to support public policies through the management in anticipation of the infinite interactive possibilities related to the post-emergency period and the promotion of change in the direction of Community Health.

## Data Availability Statement

The original contributions presented in the study are included in the article/Supplementary Material, further inquiries can be directed to the corresponding author/s.

## Author Contributions

GPT as scientific reviewer was involved in assessing the appropriateness and scientific relevance of the theoretical methodological assumptions used. MSDR dealt with the social cohesion index and the measurement, using the methodology of reference. CC and CM carried out and reviewed the links with other research and projects using the same methodology. LO has been involved in the research and use of scientific literature on the topics. All authors contributed to the article and approved the submitted version.

## Conflict of Interest

The authors declare that the research was conducted in the absence of any commercial or financial relationships that could be construed as a potential conflict of interest.

## Publisher's Note

All claims expressed in this article are solely those of the authors and do not necessarily represent those of their affiliated organizations, or those of the publisher, the editors and the reviewers. Any product that may be evaluated in this article, or claim that may be made by its manufacturer, is not guaranteed or endorsed by the publisher.

## Footnotes

^1^“Diachronic process of different typology (energetic, metabolic, dialogic) by means of which, starting from two or more elements generated by the same process (previously or at the same time), assets/configurations, in which the elements can or cannot be more distinguishable, are generated” Turchi and Gherardini, 2014a, p.141.

^2^Epistemology is defined as “the branch of general theory of knowledge that deals with problems such as the foundations, limits, nature, and conditions of validity of scientific knowledge; it is the study of the general criteria that allow us to distinguish scientific judgements from those of opinion typical of metaphysical and religious constructions, of ethical evaluations.” (Turchi and Della Torre, 2017).

^3^“Concepts of realities on the basis of which the scientific community generates knowledge products” (Turchi, 2017).

^4^“Science that has as its object of knowledge the use of the symbolic units that compose ordinary language, which gives form to discursive configurations; [it is] that cognitive apparatus that formalises the dialogical process (or discursive process)” Turchi and Gherardini, 2014b, p. 215.

^5^“Intrinsic characteristic of uncertainty that in its manifestation finds its own structure that tends to stabilise and become distinguishable and denominable in a language (except then interact with the whole and become a further element that generates uncertainty)” (Turchi and Vendramini, 2016).

^6^Ordinary language is the whole of symbolic units and rules of application that govern its use, at the basis of the discursive process.

^7^“The property of language to assume a different value every time the symbolic unit is used” (Turchi, 2009).

^8^“A finite mode of constructing reality, linguistically understood, with pragmatic value, which groups together even more enunciated (called “archipelagos of meaning”), articulated in concatenated sentences and diffused with a value of assertion of truth, aimed at generating (building)/maintaining a narrative coherence” Turchi and Della Torre, 2017, p. 91, Turchi and Orrù, 2014, p. 13.

^9^(Turchi and Orrù, 2014).

^10^By conflict it is meant the interactive setup of high fragmentation risk; here, the Veneto Region community members interact on the grounds of strongly personal aims and references, therefore with a very low/void orientation towards common and third goals. Since the personal aim interacts with the personal stance of another Community member, one excludes the other and their co-existence is not contemplated.

^11^The formula that allows to calculate Generativity (G) has been constructed as G = ΔpΔmD, in which Δp indicates the weight of the configuration; Δm indicates the moment in which the configuration is traced; and D indicates the constant, the quantum of Generativity, that is the discursive space that can be generated (in which D≥ ΔpΔm).

^12^“The intrinsic and organising property of the elements that constitute the discursive productions, to maintain congruence and uniformity in such a way that it is not possible to generate a contradiction in narrative terms, generating a configuration that, by common sense, is set as given and certain” (Turchi and Gherardini, 2014b).

^13^Collected in accordance with Italian privacy regulations.

^14^The defined goal/objective, as part of the discursive space, can modify and change content; however, the adoption of the Targeting Repertory as an observer does not change.

^15^In the absence of empirical-factual causal links, it is not possible to “predict” what will happen, but one can rely on the narrative coherence of a discursive process in order to anticipate which configurations of reality could be generated compared to the available one (Turchi and Della Torre, 2017). The anticipation of future scenarios allows the members of a community to build shared management choices to go in the direction of the common goal.

^16^“Configuration of the interactive asset of the Community in which each member, or aggregation of members of the same, can build its own dialogic node in the interactive asset and thus contribute to its development, towards the generation of Social Cohesion for the entire Community of belonging” (Turchi and Gherardini, 2014b).

^17^As for the Targeting Repertoiry, it really is a high Dialogical Weight and a medium Dialogical Moment.
